# MiR-146a alleviates lung injury caused by RSV infection in young rats by targeting TRAF-6 and regulating JNK/ERKMAPK signaling pathways

**DOI:** 10.1038/s41598-022-07346-6

**Published:** 2022-03-03

**Authors:** Zhi Huang, Xiaoxian Liu, Xi Wu, Min Chen, Wenfeng Yu

**Affiliations:** 1 Department of Interventional Radiology, the Affiliated Hospital of Guizhou Medical University, Guiyang, 550001 China; 2 Department of Medicine Intersive Care, Affiliated Hospital of Guizhou Medical University, Guiyang, 550001 China; 3 Department of Pneumology, Maternal, Child Health Hospital of Guiyang City, Guiyang, 550001 China; 4 School of Basic Medical Science, Guizhou Medical University, Guiyang, 550002 China

**Keywords:** Respiratory tract diseases, Infection

## Abstract

Respiratory syncytial virus (RSV) is a major cause of acute lower respiratory tract infection in infants and children. The present study aimed to investigate the effects of miR-146a on RSV replication and the related mechanisms. Material and methods: We pretreated A549 and HEp-2 cells and young rats with miR-146a mimic before infection with RSV. The expressions of miR-146a and RSV-F mRNA in cells and lung tissues were detected by RT-qPCR, and production of IL-1β, IL-6, IL-18, and TNF-α in bronchial alveolar lavage fluid (BALF) were determined by ELISA. The expression level of TRAF-6 and activation of the JNK/ERK/MAPK/NF-κB signaling pathway was detected by Western blotting. Results: RSV infection significantly reduced miR-146a levels in both A549 and HEp-2 cells and rat lung tissues. RSV infection resulted in accelerated growth, increased release of inflammatory cytokines, increased expression of TRAF-6, and activation of the JNK pathway in cells, and the lung inflammatory infiltration and the pathological score increased in rats. Overexpression of miR-146a targeted down-regulation of TRAF-6 expression and JNK/ERK/MAPK/NF-κB pathway induced by RSV infection, reduced the production of inflammatory cytokines IL-1β, IL-6 and TNF-α, and alleviate lung injury in young rats. We got similar results in both A549 and HEp-2 cell experiments. Conclusion: MiR-146a alleviates lung injury caused by RSV infection in young rats by targeting TRAF-6 and regulating JNK/ERK/MAPK signaling pathways.

## Introduction

Human respiratory syncytial virus (RSV) is an encapsulated, negative single-stranded RNA (ssRNA) virus, belonging to pneumonoviridae^1^. RSV is a major cause of respiratory infections in infants and young children, leading to pneumonia and bronchiolitis. In addition, severe RSV infection is associated with the development of recurrent wheezing or asthma^2^. RSV is also an important cause of respiratory tract infection in older people over 65 years old^3^. Despite ongoing progress in the development of a vaccine for RSV^4^, there is an urgent need for effective treatment strategies and screening biomarkers for RSV.

MicroRNAs (miRNAs) are a class of small non-coding RNAs of 20∼22 nt in length that usually exert their effects through directly binding to the 3′-untranslated regions (UTRs) of their target mRNAs^5^, resulting in the degradation of these mRNAs or their translational inhibition^6^. Many miRNAs have been confirmed to be associated with RSV infection. For example, microRNA 146-5p, miR-let-7c-5p, miR-221 and miR-345-5p are differentially expressed in RSV persistently infected HEp-2 cells^7^. Peripheral blood microRNAs expression is associated with infant RSV infection^8^. RSV has been found to alter microRNA expression in a cell type-specific manner, and RSV appears to manipulate gene expression in host cells by regulating the expression of miRNAs associated with interferon response^9^. Other studies have confirmed the regulation of miRNA on RSV replication: microRNA-221 regulates RSV replication in human bronchial epithelium by targeting NGF expression^10^. These evidences reveal the key role of miRNAs in RSV infection and replication.

In the previous study, miR-146a was identified as a potential biomarker of sepsis and played a role in regulating inflammation^11^. A large number of studies have confirmed that miR-146a can be used as a marker of acute lung injury, and has a regulatory effect on the inflammatory response of asthma and acute lung injury^12–14^. However, its roles in lung injury caused by RSV virus infection remain uncertain. In a study^15^ of mechanical transduction and pressure-induced inflammation in small airway epithelium, primary human small airway epithelial cells (HSAEpCs) exposed to oscillatory pressure and/or pro-inflammatory cytokine TNF-α for 12 h significantly increased IL-6, IL-8, and IL-1β cytokines secretion in a time-dependent manner. Meanwhile, the expression level of miR-146a was most significantly affected. Both regulations of miR-146a expression and silencing of TNF receptor-associated factor-6 (TRAF-6) resulted in a significant reduction in stress-induced cytokine secretion, suggesting that miR-146a targets the potential role of TRAF-6 in airway inflammation.

In this study, we infected young rats and cells with RSV virus respectively and upregulated miR-146a levels with miRNA mimics to explore the effects of miR-146a on lung injury of young rats and cell function, and attempted to explore its potential mechanism of action, providing new data support for the treatment strategy of RSV infection.

## Methods

### Cell culture and treatment

Human epidermoid cell line type 2 cells (HEp-2), and A549 cells were obtained from American Type Culture Collection (ATCC) and cultured in Dulbecco modified Eagle medium (DMEM) supplemented with 10% fetal bovine serum (FBS), 100 U/mL penicillin, and 100 μg/mL streptomycin at 37℃ in a humidified atmosphere of 5% CO_2_. Medium and all the other culture reagents were purchased from Gibco (USA). A549 and HEp-2 cells were transfected with 50 nM hsa-miR-146a mimic or miRNA negative control (GenePharma, Shanghai, China) using Lipofectamine 2000 (Invitrogen) according to the manufacturer’s instructions. pcDNA3.1 plasmids were inserted with the sequences of TRAF-6 for overexpressing TRAF-6, and pcDNA3.1 as control. After transfection for 6 h, cells were washed with ice-cold PBS twice and then cultured for an additional 24 h. The long strain of RSV was obtained from ATCC and propagated in HEp-2 cells. Cells were pretreated with miR-146a mimic or miRNA negative control at the indicated concentrations for 24 h before being infected with RSV at a multiplicity of infection (MOI) of 3^16^. The A549 and HEp-2 cells were divided into four groups, respectively: CON group (miRNA negative control transfection only), RSV group (RSV infection and miRNA negative control transfection), miR-146a group (miR-146a mimic transfection only), and RSV + miR-146a group (RSV infection and miR-146a mimic transfection). At 8 h post infection, supernatants were collected for ELISAs, or the cells were collected for RNA or protein extraction.

### CCK‑8 assay

A549 and HEp-2 cells were plated into 96-well plates at a density of 1 × 10^3^/well. Twenty-four hours later, the cultural media were replaced with media. After culturing for 0, 24, 48, 72, and 96 h, 10 μL of CCK-8 solution (Beyotime Institute of Biotechnology, China) was added, and the cells were cultured for another hour before absorbance at 450 nm was measured using a microplate reader (BioTek Instruments, USA). The experiment was repeated in triplicate with 3 replicates.

### Reverse transcription‑quantitative polymerase chain reaction (RT‑qPCR) analysis

Total cellular RNA was extracted from cultured cells or bronchial alveolar lavage fluid (BALF) with TRIzol reagent (Invitrogen). To analyze mRNA and miRNA expression, 1 μg of RNA was reverse transcribed to cDNA with a PrimeScript RT Reagent Kit (TaKaRa), and quantitative real-time PCR was then performed with SYBR Green qPCR Master Mix (TaKaRa) and miRcute miRNA qPCR Detection Kit (Tiangen Biotech Co., Ltd.). The specific primers were synthesized by GenePharma (China). GAPDH (forward 5ʹ-TCACCAGGGCTGCTTTTAAC-3ʹ, reverse 5ʹ-GACAAGCTTCCCGTTCTCAG-3ʹ) and U6 (forward 5ʹ-GCTTCGGCAGCACATATA CTAAAAT-3ʹ, reverse 5ʹ-CGCTTCACGAATTTGCGTGT CAT-3ʹ) were served as the housekeeping gene. Sequences of the primers used for RT-qPCR were as follows: miR-146a, forward 5ʹ-CAACACCAGTCGATGGG CTGT-3ʹ, reverse 5ʹ-CCCAUGGAAUUCAGUUCUC AUU-3ʹ; TRAF-6, forward 5′-CCTTTGGCAAATGTCATCTGTG-3ʹ, reverse 5′-CTCTGCATCTTTTCATGGCAAC-3ʹ. All reactions were performed in triplicate, and the data were analyzed with the double delta cycle threshold (CT) method of relative quantification.

### ELISA

To assess the production of cytokines, cell supernatants and BALF were collected. Enzyme-linked immunosorbent assay (ELISA) kits for human and rat IL-1β, IL-6, IL-18, and TNF-α were purchased from Boster Biological Technology Co. Itd as previously described^17^. Levels of cytokines were determined with commercial ELISA kits according to the manufacturer’s instructions. The absorbance at 490 nm was read on an ELISA plate reader. The experiment was repeated in triplicate with 3 replicates.

### Dual-luciferase reporter assay

The experiment was performed as previously reported^18^. Wild sequence (WT-TRAF-6) and mutant sequences (MUT-TRAF-6) were designed and synthesized by GenePharma. Sequences were inserted into a luciferase reporter vector (pGL3-Basic). Vectors were co-transfected with miR-146a mimic or miRNA mimic NC into HEK293T cells. Cells were lysed with 100 μL of lysis buffer in a shaking bed at room temperature for 20 min. The cell suspension was incubated with luciferase solution (Promega) before the intensity of Firefly luciferase was determined. Stop&Glo reagent I from Promega was fully mixed with cell suspension, and then the intensity of Renilla luciferase was measured. The activity of Renilla luciferase was considered as an internal control. The relative luciferase activity shall be the ratio of firefly luciferase intensity and Renilla luciferase intensity. Triplicate wells were set for each group.

### Western blotting

Western blotting was performed using the standard SDS-PAGE separation technique as previously reported^18,19^. The harvested cells were disrupted with RIPA cleavage buffer (Cell Signaling Technology). Proteins were extracted from exosomes using a Total Exosome Protein Isolation Kit (Invitrogen, Carlsbad, CA, USA) following the instructions supplied. The lysates were collected after centrifugation, and the protein concentration was quantified with BCA kit (Thermo Fisher Scientific). 50 mg of protein was added to sodium dodecyl sulfate polyacrylamide gel electrophoresis (SDS-PAGE) and then were transferred onto polyvinylidene difluoride (PVDF) membranes (Millipore, USA). The membranes were incubated overnight at 4 °C with antibodies against phosphor-JNK (ab124956), phospho-MAPK (ab195049), phospho-ERK (ab201015), phospho-NF-κB (ab76302), TRAF-6 (ab33915) and GAPDH (ab8245) purchased from Abcam. Subsequently, the membranes were incubated with a secondary antibody for 1.5 h at room temperature. Immunoreactive protein bands were detected using an Odyssey scanning system (USA), quantified by Image J, and normalized to the corresponding amount of total protein. The experiment was repeated in triplicate with 3 replicates.

### Animal models

Thirty female Wister rats (5 ~ 6 weeks old) were obtained from the Shanghai Laboratory Animal Company (China). The current study received ethical approval from the Animal Care and Use Committee of Affiliated Hospital of Guizhou Medical University, and experiments and animal care were performed according to the approved protocols. Rats were randomly divided into three groups (n = 10) as follows: CON group: rats were mock-infected + miRNA mimic negative control; RSV group: RSV + miRNA mimic negative control; and RSV + miR-146a group: RSV + miR-146a mimic. The rats were treated with a nasal drip of interferences 24 h before RSV infection. For RSV infection, rats were anesthetized and then intranasally inoculated with 5 × 10^5^ PFU RSV in a total volume of 20 μL. BALFs were harvested, and the lungs were collected when rats showed pneumonia-related symptoms.

### Rat appearance status rating

The general state of the rats, including diet, water intake, hair activity, mental state, body weight, and respiratory rate, was observed every day, and the relevant data were recorded. The general state assessment of rats was divided into four grades: a. Excellent: good mental state, flexible reaction, quick behavior and activity, white and shiny hair, normal diet and water breathing rate, and gradually increased body weight; B. Good: general mental state, smooth hair color, flexible reaction, stable breathing, slightly reduced dietary water, bodyweight close to normal unchanged; C. Not well: poor mental state, loss of luster, listlessness, less movement, increased respiratory rate, decreased dietary water volume, slight weight loss; D. Poor: lethargy, slow reaction, slow action, a severe reduction in dietary water volume, severe weight loss.

### Hematoxylin and eosin (HE) staining

After fixation with paraformaldehyde and decalcified in 10% EDTA, the lung tissues were embedded in paraffin and sectioned into a 5-μm-thick section. The selected sections were deparaffinized in xylene, rehydrated through a graded series of ethanol washes, and followed by hematoxylin and eosin (HE) staining. The sections were washed with a weak acid solution for 1 min and washed with distilled water for 1 min. The sections were dehydrated and transparent, and finally observed under a light microscope.

### Lung injury assessment

The weight of rats in each group was measured by electronic balance, and then the lung tissue was measured by lung weight, and the lung index was calculated as rat lung weight (g)/rat body weight (g) × 100%.

Lung histopathological score index standard:

A.: Area of parapbronchial inflammation injury (ratio of sites): intact trachea structure and no abnormality around was marked as 0; The area of peribronchial inflammatory infiltration below 25% was marked as 1 point; There were many inflammatory cells infiltrating, and the damaged area between 25 and 75% were marked as 2 points; Inflammatory damage of more than 75% of the area was marked as 3 points;
B.: Changes in the number and structure of inflammatory cells around the bronchus: no inflammatory cells or occasional lymphocytes around the bronchus were marked as 0; Slight edema with frequent discontinuities in the bronchial ring was marked as 1 point; Moderate edema around the bronchus, annular structure nearly complete or crescent ring was denoted as 2 points, equivalent to the thickness of 5 cells; The number of lymphocytic infiltrates increased, and severe complete ring formation was observed, with thickness close to 5 ~ 10 cells were marked as 3 points;
C.: Amount of inflammatory exudation in the alveolar cavity: the alveolar structure was clear and complete, and no inflammatory factor was marked as 0; Mild inflammatory cell infiltration, only accounted for less than 25% of the alveolar closed cavity marked as 1 point; When 25% of the cavity is closed, it is severe and is recorded as 2 points;
D.: Capillary infiltration range (percentage of position) no abnormality was found in the surrounding capillary structure, which was marked as 0 points; When the infiltration area less than 10% was recorded as 1 point; The infiltration area between 10 and 50% was 2 points; When the area of infiltration of perivascular lung tissue increased to more than 50%, it was marked as 3 points;
E.: Substantial pneumonia: no substantial pathological changes were marked as 0; mild consolidation or speckled substantial infiltration of pneumonia was marked as 1 point; severe pulmonary parenchymal lesions, substantial infiltration with enlarged spots or fusion marked as 2 points;

The histopathological score (0 ~ 26 points) was calculated as A + 3 × (B + C) + D + E.

### Plaque assay

The viral titer was determined by the plaque assay as previously reported^20^. HEp-2 and A549 cells were plated into 12-well plates and allowed to grow into a monolayer. Virus stocks, cell-free medium, or lung homogenates from the rats were serially diluted tenfold at a volume of 200 μL and then incubated with HEp-2 cells. After 1 h incubation, the supernatants were removed, and the cells were overlaid with 1 mL of 1% (wt/vol) methylcellulose containing 50% (vol/vol) DMEM and 2% (vol/vol) FBS and cultured for another 5 days before the overlay medium was removed. Cells were fixed and stained with 2% (wt/vol) crystal violet in 20% (vol/vol) ethanol. The plaques were observed, and plaques in wells containing 30 ~ 100 plaques were counted. Viral titers were calculated using the following formula: viral titer (PFU/mL) = plaques × dilution × 5.

### Statistical analysis

All data were analyzed using SPSS 17.0 software (USA). Data are presented as the means ± standard deviation (SD). Statistical mapping was performed using Prism GraphPad 8.0 software. Comparisons between two groups were performed using the Student’s t-test and ANOVA with post hoc tests. *P* < 0.05 indicated significance.

### Ethics approval and consent to participate

The study was carried out in compliance with the ARRIVE guidelines. The animal experiments involved in this study have been approved by the Ethics Committee of the Affiliated Hospital of Guizhou Medical University.

### Consent for publication

All the authors agreed to publish the manuscript.

## Results

### Overexpression of miR-146a ameliorates the inhibition of RSV infection on cell growth by targeting TRAF-6

To explore the antagonistic effect of miR-146a on RSV, we transfected A549 and HEp-2 cells with miR-146a mimic and RSV simultaneously, and detected the cellular activity, and calculated the growth inhibition rate of the two types of cells.

**Figure 1:**
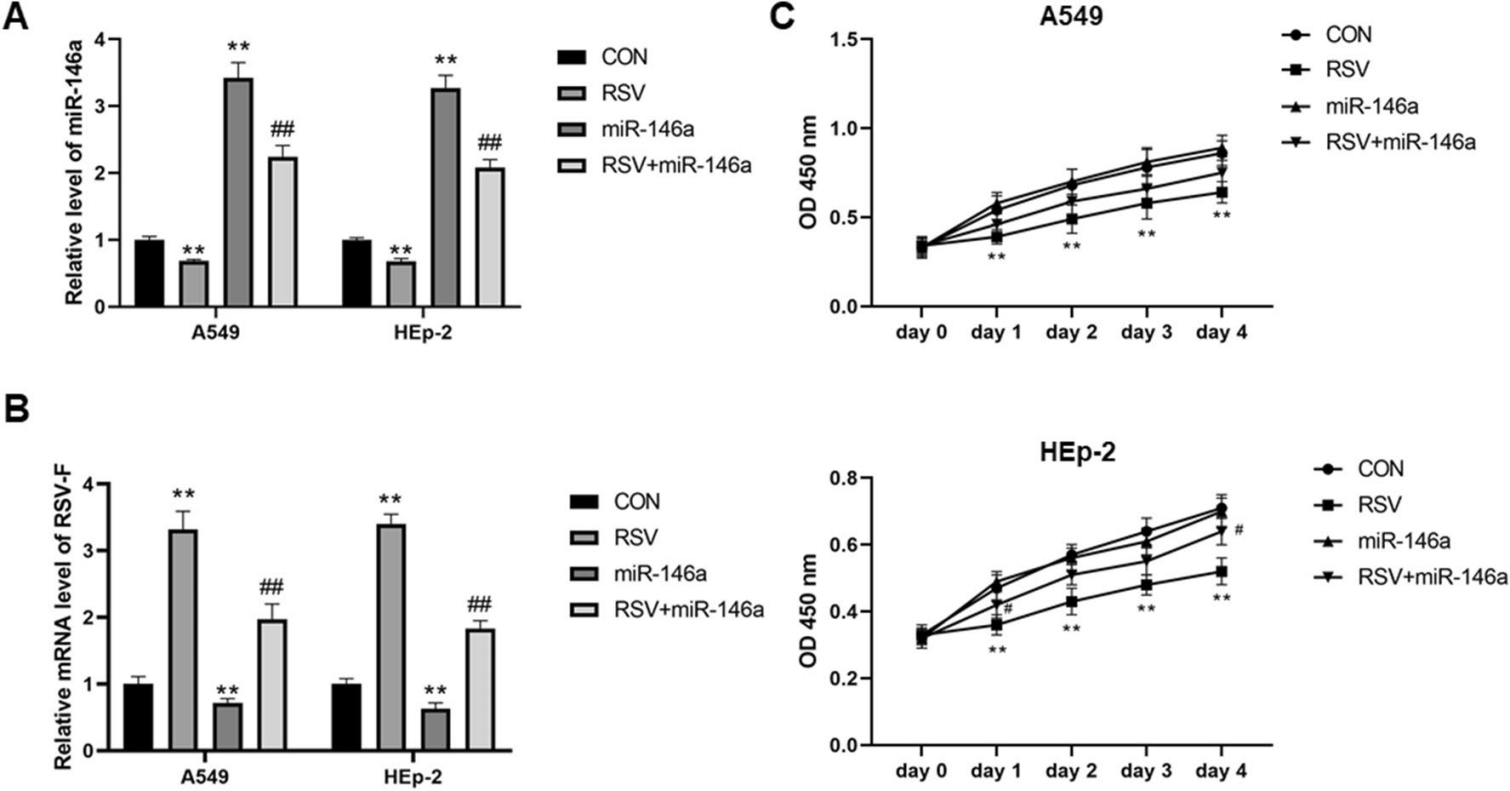
Overexpression of miR-146a slow down the accelerated cell growth induced by RSV infection. (**A**) RSV infection increased the expression of RSV-F in both A549 and HEp-2 cells, and overexpression of miR-146a significantly reduced the expression level of RSV-F mRNA; (**B**) After RSV infection, miR-146a levels were significantly decreased in both A549 and HEp-2 cells, and miR-146a mimics effectively increased the expression levels of miR-146a in the two cells; (**C**) Effects of miR-146a overexpression and RSV infection on A549 and HEp-2 cell proliferation; **P < 0.01, compared with the CON group; ##*P* < 0.01, compared with the RSV group.

Real-time quantitative PCR was used to detect the expression levels of RSV-F mRNA and miR-146a in both A549 and HEp-2 cells to verify the grouping. RSV infection significantly increased the expression level of RSV-F mRNA in both A549 and HEp-2 cells (*P* < 0.01), and transfection with miR-146a mimics also effectively increased the expression level of miR-146a in the two kinds of cells compared with negative control transfection (*P* < 0.01, Fig. 1A,B). Notably, compared with the CON group, overexpression of miR-146a significantly reduced the expression level of RSV-F mRNA, and RSV infection significantly reduced miR-146a expression levels in both A549 and HEp-2 cells (*P* < 0.01, Fig. 1A,B). RSV infection significantly decreased the growth of A549 cells and HEp-2 cells (*P* < 0.01), while increasing the growth inhibition rate (*P* < 0.01, Fig. 1C). Overexpression of miR-146a could significantly ameliorate the inhibited growth rate and decreased inhibition rate induced by RSV infection (*P* < 0.05, Fig. 1C).

**Figure 2:**
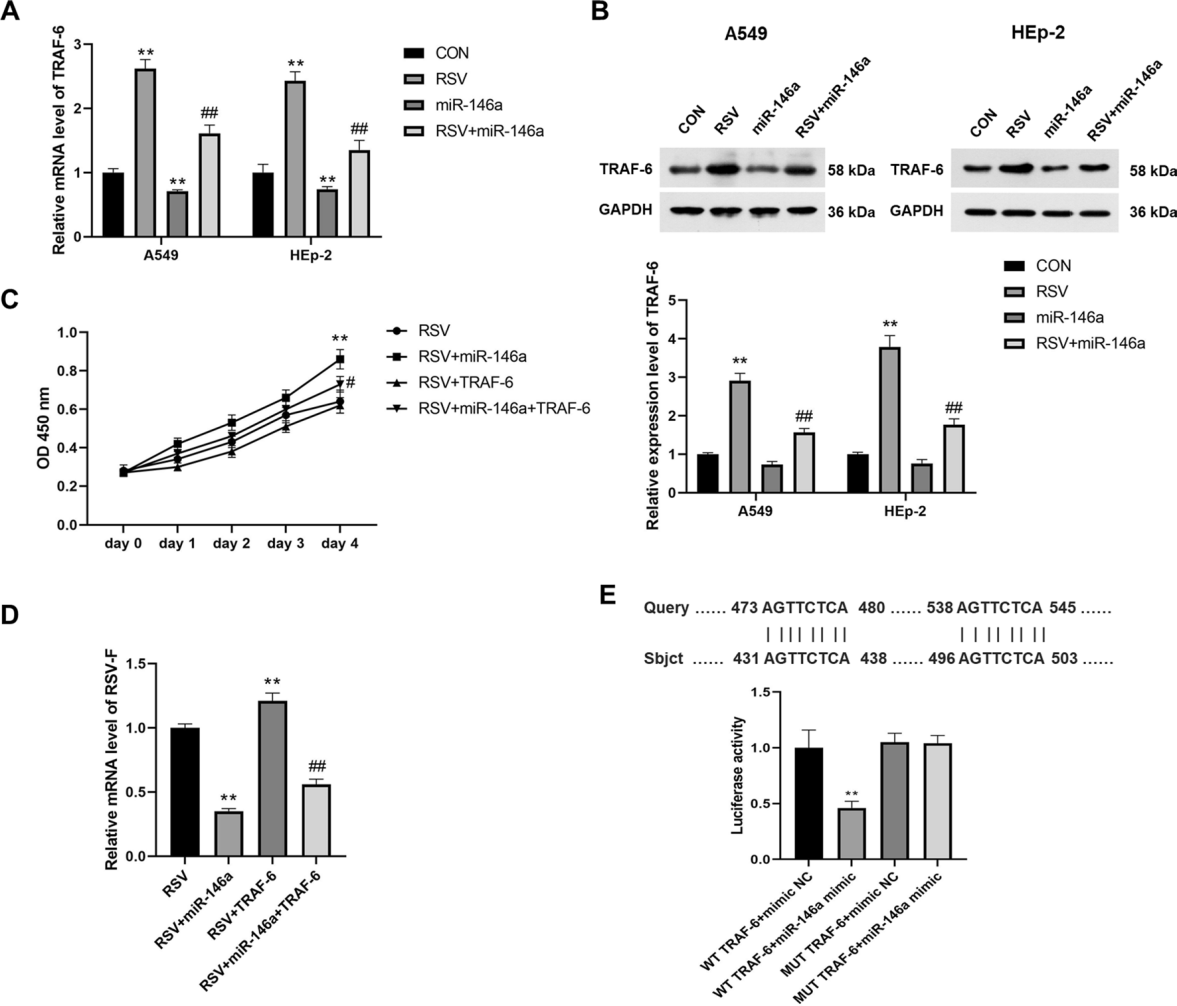
RSV infection significantly reduced the level of miR-146a in cells and miR-146a negatively regulated TRAF-6. (**A**) After RSV infection, the mRNA expression level of TRAF-6 in both A549 and HEp-2 cells was significantly increased, and the overexpression of miR-146a significantly inhibited the mRNA expression of TRAF-6; (**B**) After RSV infection, the protein expression level of TRAF-6 in both A549 and HEp-2 cells was significantly increased, and the overexpression of miR-146a significantly inhibited the protein expression of TRAF-6; **P < 0.01, compared with the CON group; ##*P* < 0.01, compared with the RSV group. (**C**) Overexpression of TRAF-6 reversed the accelerated RSV-infected A549 cell growth induced by transfection with miR-146a mimics; (**D**) Overexpression of TRAF-6 reversed the decreased RSV-F mRNA expression induced by transfection with miR-146a mimics; **P < 0.01, compared with the RSV group; #*P* < 0.05, ##*P* < 0.01, compared with the RSV + miR-146a group. (**E**) The dual luciferase gene report showed that miR-146a has a targeting relationship with TRAF-6.

In addition, we detected the mRNA and protein expression of TRAF-6 in each group, and found that RSV infection significantly increased the mRNA and protein expression of TRAF-6 in both A549 and HEp-2 cells (*P* < 0.01), while overexpression of miR-146a significantly decreased the expression level of TRAF-6 (*P* < 0.01, Fig. 2A,B). Overexpression of miR-146a significantly reduced the expression level of TRAF-6 in cells elevated with RSV infection compared with cells infected only with RSV (*P* < 0.01, Fig. 2A,B).

To verify the targeting effect of miR-146a on TRAF-6, we transfected RSV-infected A549 cells with both miR-146a mimics and TRAF-6 overexpression plasmid. Overexpression of TRAF-6 can slow down the growth acceleration of RSV-infected cells caused by miR-146a (*P* < 0.05, Fig. 2C). Moreover, overexpression of TRAF-6 significantly increased the expression of RSV-F mRNA in A549 cells (*P* < 0.01), and reversed the decreased expression of RSV-F mRNA caused by transfection with miR-146a mimics (*P* < 0.01, Fig. 2D). The dual-luciferase gene report revealed the interaction sites between miR-146a and TRAF-6, confirming the interaction relationship between the two (*P* < 0.01, Fig. 2E).

### Overexpression of miR-146a inhibited RSV-induced inflammatory cytokine release and JNK/ERK/MAPK pathway activation

**Figure 3:**
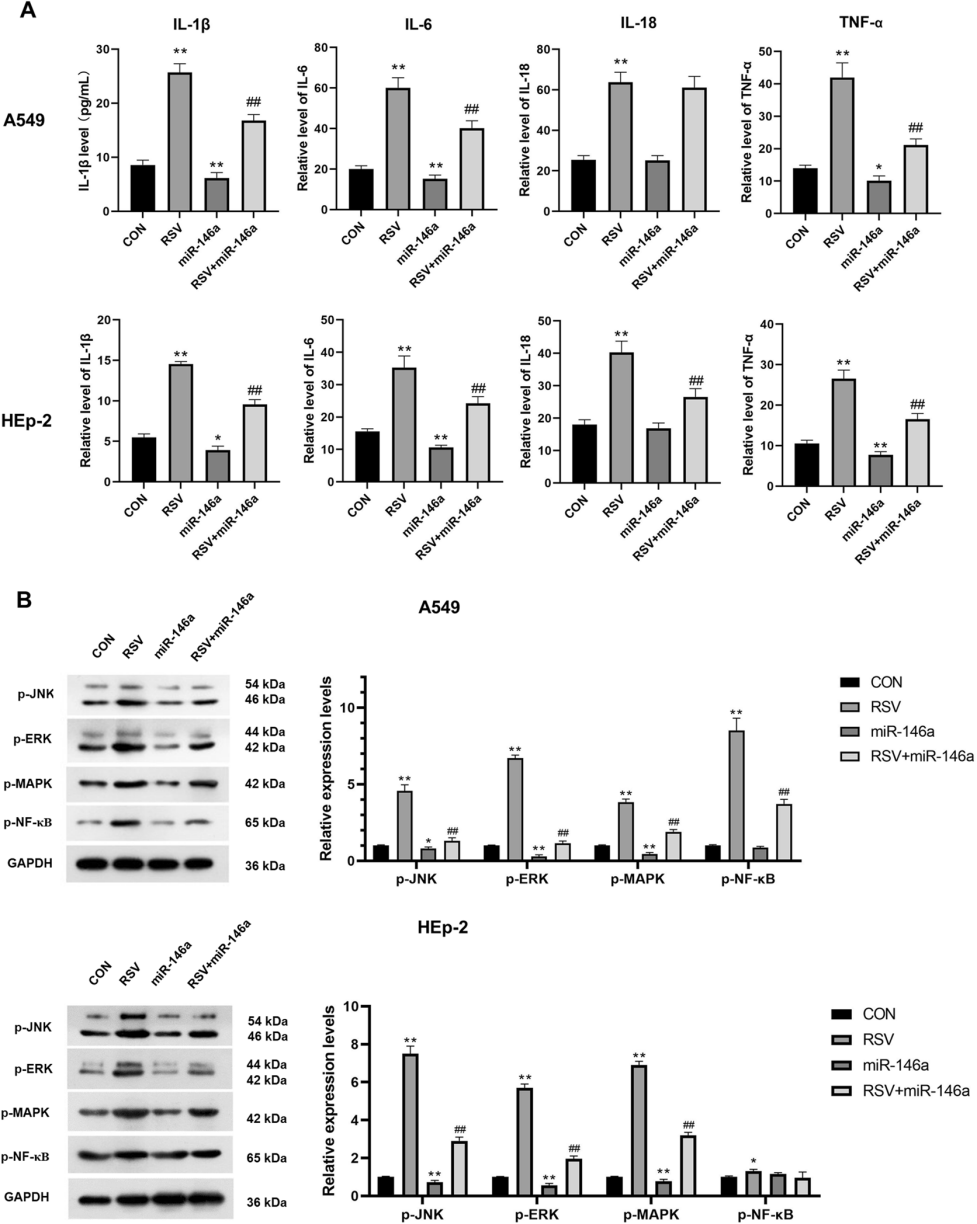
Overexpression of miR-146a inhibits RSV-induced inflammatory cytokines release and JNK/ERK/MAPK/NF-κB pathway activation in both A549 and HEp-2 cells. (**A**) Levels of inflammatory cytokines, including IL-1β, IL-6, IL-18 and TNF-α, in A549 and HEp-2 cells in each group; (**B**) Protein expression levels of JNK, MAPK, ERK and NF-κB in A549 and HEp-2 cells in each group; *P < 0.05, **P < 0.01, compared with the CON group; ##*P* < 0.01, compared with the RSV group.

We used ELISA to detect the release of inflammatory cytokines in each group. RSV infection significantly increased levels of inflammatory cytokines in both A549 and HEp-2 cells, including IL-1β, IL-6, IL-18, and TNF-α, compared with the CON group (*P* < 0.01, Fig. 3A). Overexpression of miR-146a significantly reduced IL-1β, IL-6, and TNF-α levels in both A549 and HEp-2 cells compared to the CON group (*P* < 0.01, Fig. 3A). In HEp-2 cells, the levels of IL-1β, IL-6, IL-18, and TNF-α in the RSV and miR-146a transfected group were significantly lower than those in the RSV group, while only IL-1β, IL-6, and TNF-α levels were significantly reduced in A549 cells (*P* < 0.01, Fig. 3A).

Phosphorylation of JNK/ERK/MAPK/NF-κB signaling pathway related proteins was detected by Western Blot. RSV infection significantly increased phosphorylation levels of the c-Jun n-terminal kinase (JNK), MAPK, ERK, and NF-κB in both A549 and HEp-2 cells compared with the CON group (*P* < 0.05 or *P* < 0.01, Fig. 3B). The phosphorylation levels of JNK, ERK, and MAPK were significantly decreased in A549 and HEp-2 cells when miR-146a was overexpressed compared with the CON group (*P* < 0.05 or *P* < 0.01, Fig. 3B). In A549 cells, overexpression of miR-146a significantly reduced the increased phosphorylation levels of JNK, MAPK, ERK, and NF-κB by RSV infection (*P* < 0.01, Fig. 3B), while in HEp-2 cells, there was no significant difference in NF-κB phosphorylation between RSV + miR-146a group and RSV group (*P* > 0.05, Fig. 3B).

### Overexpression of miR-146a may alleviate lung injury caused by RSV infection in young rats through TRAF-6 or JNK/ERK/MAPK pathway

**Figure 4:**
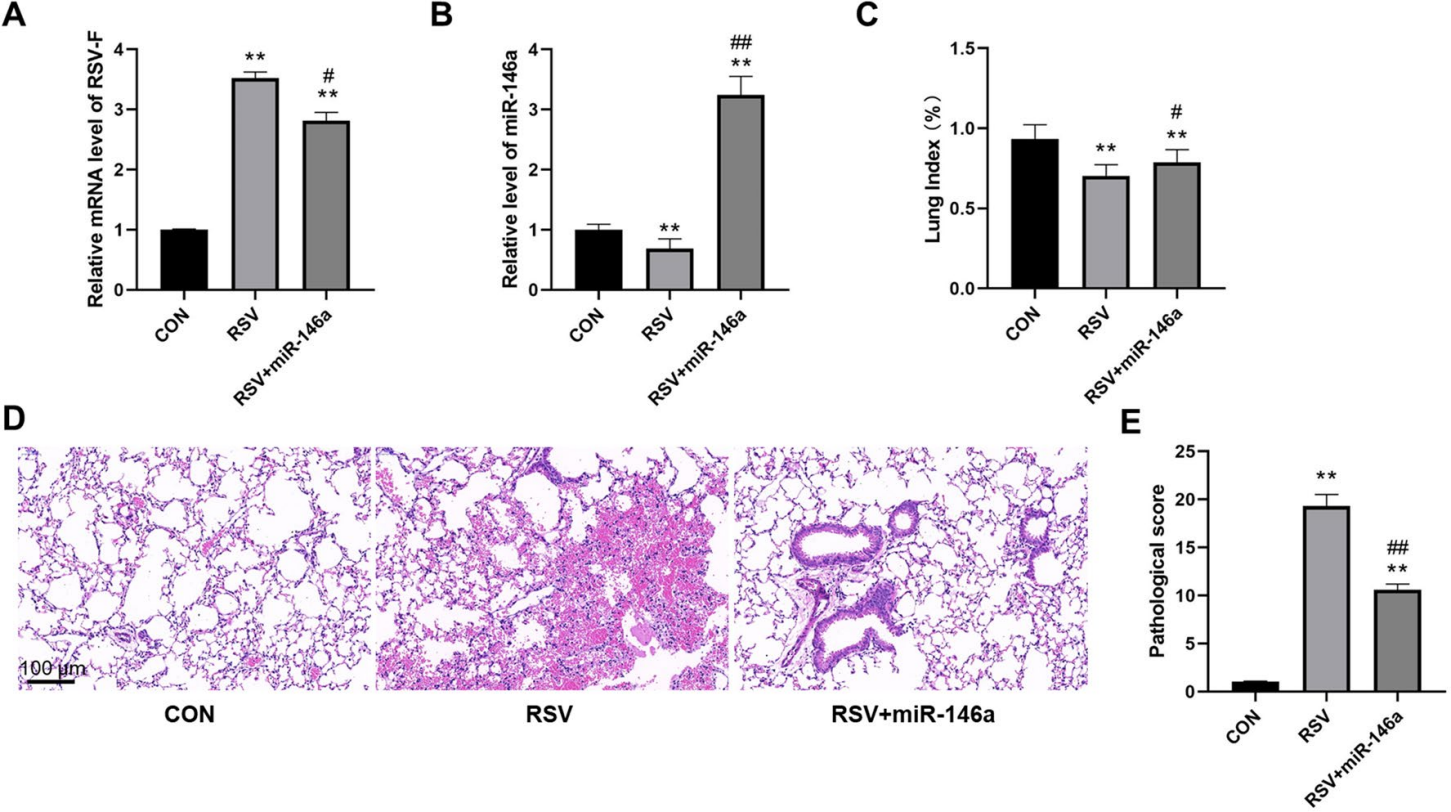
Overexpression of miR-146a significantly alleviated lung injury caused by RSV infection in rats. (**A**) Overexpression of miR-146a significantly reduced the increased expression of RSV-F mRNA in BALF of rats induced by RSV infection; (**B**) The level of miR-146a in BALF was significantly decreased in rats infected with RSV; (**C**) Overexpression of miR-146a significantly increased the decreased lung index induced by RSV infection in rats; (**D**) HE staining of rat lung tissues showed that overexpression of miR-146a significantly improved inflammatory infiltration induced by RSV infection; (**E**) Overexpression of miR-146a significantly reduced the pathological score of rats induced by RSV infection; **P < 0.01, compared with the CON group; #*P* < 0.05, ##*P* < 0.01, compared with the RSV group; n = 10.

We detected the expression levels of RSV-F mRNA and miR-146a in BALF of rats in each group by real-time quantitative PCR. RSV-F mRNA level in the RSV group was significantly increased compared with the CON group (*P* < 0.01), and the RSV + miR-146a group was significantly decreased compared with the RSV group (*P* < 0.01, Fig. 4A). Compared with the CON group, the expression level of miR-146a the in RSV group was significantly decreased (*P* < 0.01), and the expression level of miR-146a in the RSV + miR-146a group was significantly increased compared with the RSV group (*P* < 0.01, Fig. 4B).

In addition, the appearance and lung pathology of rats in each group were observed. The appearance status of rats was shown in Table 1. The status of rats in the RSV transfection group was significantly worse, and the overexpression of miR-146a could significantly improve the status of rats. Compared with the CON group, the lung index of RSV-infected rats was significantly decreased (*P* < 0.01), and overexpression of miR-146a significantly increased lung index compared with the RSV group (*P* < 0.05, Fig. 4C). HE staining showed that there was obvious infiltration of inflammatory cells around the alveolar wall in the RSV infected group compared with the CON group (Fig. 4D). Compared with the RSV group, overexpression of miR-146a significantly reduced peri-airway inflammation (Fig. 4D). RSV infection significantly increased the pathological score of lung tissues in rats, and overexpression of miR-146a significantly reduced the RSV-induced score increase (*P* < 0.01, Fig. 4E). All above, overexpression of miR-146a can significantly alleviate lung injury caused by RSV infection in rats.

**Table 1:** Appearance status rating of mice in each group.

CON		RSV		RSV + miR-146a	
No	Rating	No	Rating	No	Rating
1	A	1	C	1	B
2	A	2	D	2	D
3	A	3	C	3	B
4	A	4	C	4	C
5	A	5	C	5	B
6	B	6	D	6	C
7	A	7	C	7	C
8	A	8	D	8	B
9	A	9	D	9	D
10	B	10	C	10	C

**Figure 5:**
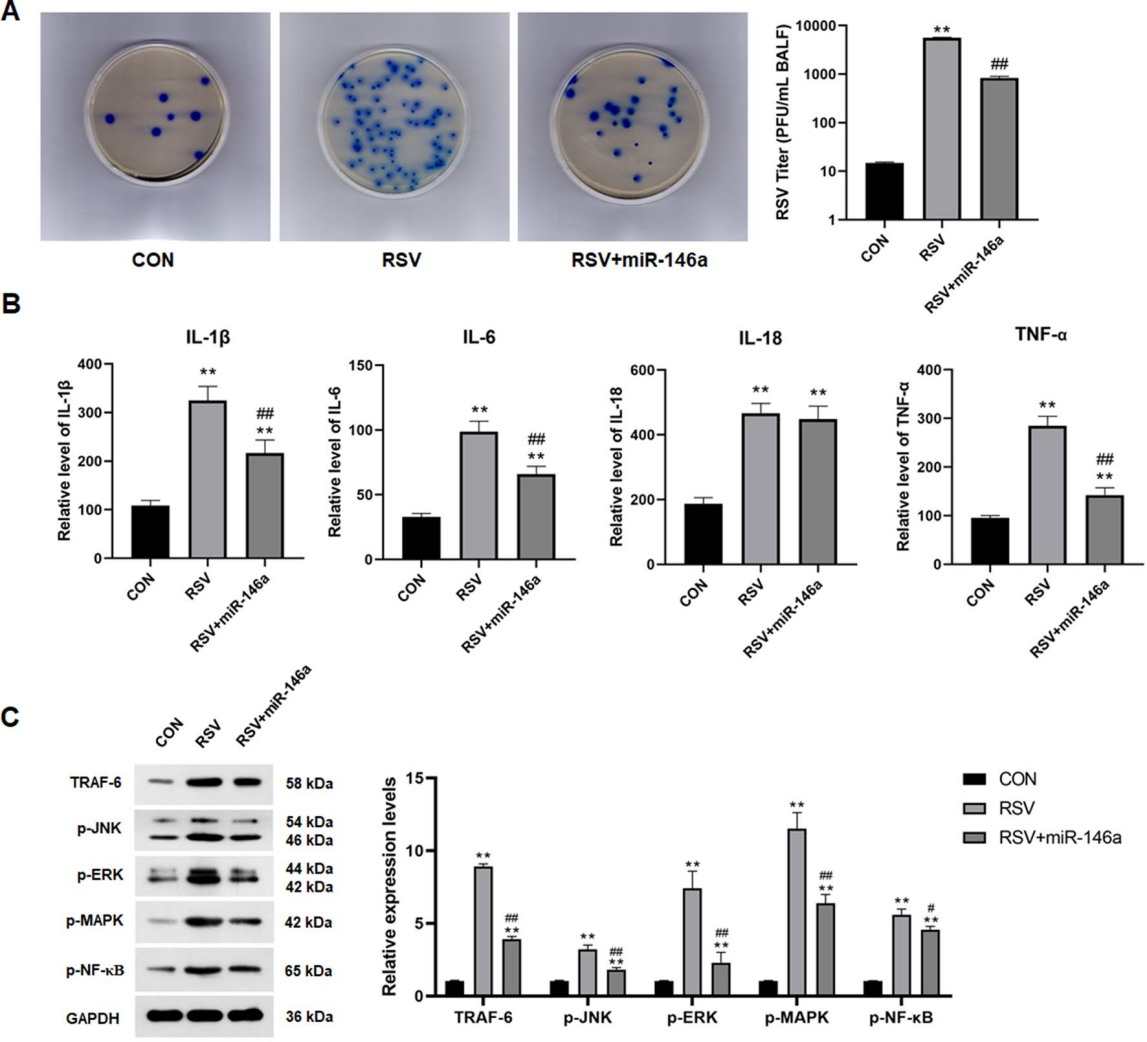
Overexpression of miR-146a inhibits RSV-induced inflammatory cytokines release and JNK/ERK/MAPK/NF-κB pathway activation in BALF of rats. (**A**) Overexpression of miR-146a significantly reduced RSV titer in rat BALF; (**B**) Levels of inflammatory cytokines, including IL-1β, IL-6, IL-18 and TNF-α, in BALF of rats in each group; (**B**) Protein expression levels of JNK, MAPK, ERK and NF-κB in BALF of rats in each group; **P < 0.01, compared with the CON group; #*P* < 0.05, ##*P* < 0.01, compared with the RSV group; n = 10.

RSV titer of BALF in rats was detected by plaque assay. Overexpression of miR-146a significantly reduced the RSV viral titer of BALF in RSV-infected rats (*P* < 0.01, Fig. 5A). RSV infection significantly increased the release of inflammatory cytokines in BALF of rats, including IL-1β, IL-6, IL-18 and TNF-α (*P* < 0.01, Fig. 5B). Overexpression of miR-146a significantly reduced the increase of IL-1β, IL-6 and TNF-α in BALF induced by RSV (*P* < 0.01, Fig. 5B). Western Blot results showed that RSV infection significantly increased the expression of TRAF-6 in BALF of rats, while overexpression of miR-146a significantly reduced the expression of TRAF-6 induced by RSV (*P* < 0.01, Fig. 5C). RSV infection also significantly increased phosphorylation levels of JNK, ERK, MAPK and NF-κB in BALF in rats (*P* < 0.01, Fig. 5C). Overexpression of miR-146a in RSV-infected rats significantly reduced the phosphorylation levels of JNK, ERK, MAPK and NF-κB in BALF (*P* < 0.05 or *P* < 0.01, Fig. 5C).

## Discussion

**Figure 6:**
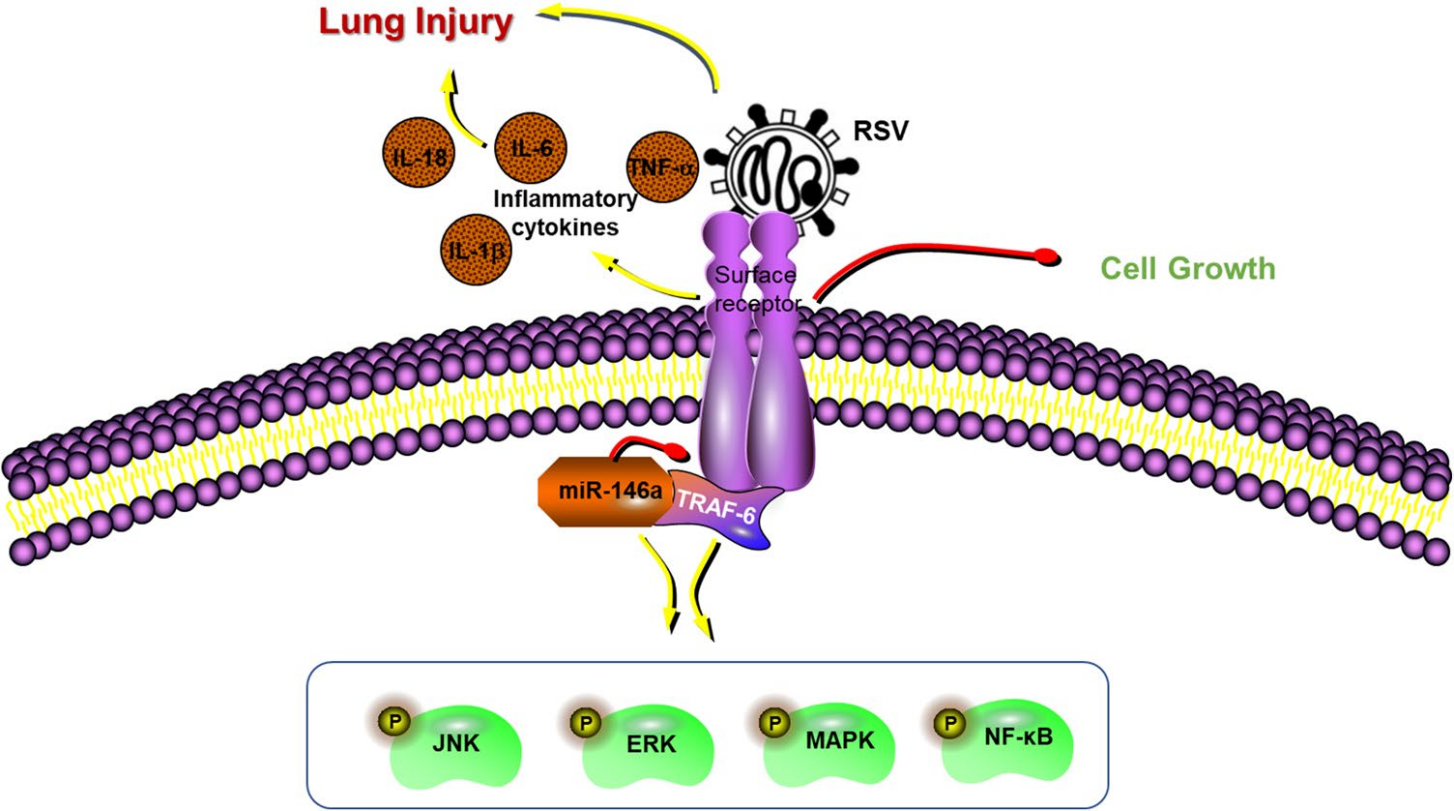
A Schematic diagram of the results of the study.

In developed countries, RSV is the leading cause of lower respiratory tract infections in infants, children under 5 years of age and immunocompromised older adults^4^. Despite many efforts to develop vaccines or drugs for RSV, there is currently a lack of specific treatment or prevention measures, possibly because the mechanism of the host response to RSV infection has not been elucidated^21^. In this study, in addition to obvious inflammatory infiltration and damage in lung tissues and significantly increased levels of inflammatory cytokines in BALF, miR-146a levels in lung tissues were significantly decreased after RSV infection in young rats. Overexpression of miR-146a in lung tissues of young rats significantly alleviated lung injury caused by RSV infection and reduced the level of inflammatory cytokines in BALF. We obtained similar results in both A549 and HEp-2 cells and confirmed the targeted negative regulation of miR-146a on TRAF-6 and its potential impact on JNK/ERK/MAPK /NF-κB signaling pathway activation. A schematic diagram is shown in Fig. 6.

Airway inflammation is an important factor leading to airway hyperresponsiveness and respiratory dysfunction in asthma. Cytokines may be involved in the occurrence and development of airway inflammation by coordinating the recruitment of airway inflammatory cells and changing the structural integrity of the airway^22^. Alveolar macrophages obtained from BALF from RSV-infected infants and adult transplant recipients co-express RSV surface glycoproteins, IL-1β, and cytoplasmic TNF-α, suggesting a local immune-regulatory and antigen-presenting role^23^. IL-6 levels were significantly higher in BALF in infants ventilated with severe RSV bronchiolitis^24^ and lower in the respiratory tract of infants with milder infection^25^. There is also an increase in nasal IL-18 concentrations and the number of IL-18-positive cells in children with RSV bronchiolitis compared to those with upper respiratory tract infection (URTI)^26^. In our study, RSV infection significantly increased the levels of inflammatory cytokines, including IL-1β, IL-6, IL-18 and TNF-α, in BALF of rats and both types of cells, which is consistent with the above results.

In addition to the increased release of inflammatory factors, RSV infection is clinically presented as paraphilia and peribronchial inflammatory infiltration, emphysema, segmentary or lobar atextention, and spotty or patchy exudation. Our animal experiments also found emphysema and bronchial inflammatory infiltration caused by RSV infection in rats, which was consistent with its clinical characteristics. After infection with RSV, the proliferation of both A549 and HEp-2 cells decreased significantly. In previous studies, RSV infection of A549 cells resulted in slow cell proliferation^27^, and the study on the cell cycle confirmed that RSV infection blocked A549 cells in the G1 phase^28^.

MiRNAs are endogenous, small non-coding RNAs that inhibit gene expression by binding the 30-untranslated regions (3’-UTRs) of target mRNAs. Viral infection (especially with RNA viruses) can subvert cellular microRNA expression, potentially to the benefit of the virus. Several studies have shown that RSV affects miRNAs expression, a property associated with the ability of this natural compound to regulate inflammation and inflammatory diseases. Numerous in vitro and in vivo studies have confirmed that miR-146a plays a critical role in airway inflammation. RT-PCR data showed that the level of miR-146a in lung tissues of asthmatic mice was higher than that of the control group, and the level of miR-146a in BALF of asthmatic children was higher than that of the control group^14^. MiR-146a significantly inhibited inflammatory cell infiltration in BALF, reduced the level of Th2 cytokines, and inhibited ovalbumin-induced airway hyperreactivity^29^. Fang et al. demonstrated that small extracellular vesicles derived from mesenchymal stromal cells can at least partially prevent group 2 innate lymphoid cells dominating allergic airway inflammation by delivering miR-146a-5p^30^.

Our study found that RSV infection significantly reduced the expression level of miR-146a in rat lung tissue and two types of cells, which was not found in previous studies. Deng et al.^31^ found that human nasal epithelial cells (hNECs) virus titer increased continuously 24 ~ 72 h after infection with H3N2 virus, and their previous studies confirmed that hNECs are highly permissive cells of respiratory viruses including influenza A virus (IAV) and RSV. HSAEpCs exposed to oscillatory pressure and/or pro-inflammatory cytokine TNF-α for 12 h significantly decreased the expression level of miR-146a^15^. These similar studies may support our results. To our surprise, overexpression of miR-146a in both tissues and cells significantly reduced RSV-induced tissue damage, cell growth inhibition and inflammatory infiltration. The anti-inflammatory effects of miR-146a have been demonstrated in a variety of airway inflammation, as we described in the previous text. In addition, miR-146a has been recognized as an anti-inflammatory factor in other cells and tissues. MiR-146a has been proposed to be an anti-inflammatory miRNA in peritoneal macrophages^32^, bronchial epithelial cells^33^, bone marrow-derived mesenchymal stem/stromal cells^34^, astrocytes^35^, BV2 microglial cells^36^ and human brain microglial cells^37^. Moreover, miR-146a targeting TRAF-6 has also been shown to play an anti-inflammatory role in a variety of diseases and studies. The results from computational miRNA target prediction algorithms revealed many more potential targets of miR-146a, but the TRAF-6 gene yielded a particularly high score^38^. Luciferase reporter assays have shown that miR-146a may combine with the 3’-UTR mRNA of TRAF-6, then downregulate the protein expression of TRAF-6^39,40^. It has been demonstrated that spinal nerve ligation (SNL) induced miR-146a-5p upregulation in the spinal cord. Intrathecal miR-146a-5p mimic attenuated SNL-induced mechanical allodynia and decreased spinal TRAF-6 expression^41^. In an in vitro hNECs model, miR-146a was overexpressed with miRNA mimics prior to H3N2 infection and reduced TRAF-6 transcripts were detected, demonstrating that H3N2-induced miR-146a can specifically target and regulate TRAF-6 expression^31^. On the other hand, miR-146a has been shown to play an important role in targeting TRAF-6 in T cells^42,43^. Our study found that overexpression of miR-146a leads to a reduction in TRAF6 translation in rats’ BALF, possibly due to the role of T cells in the respiratory tract. Our subsequent studies will further investigate specific cell types in BALF. Both regulations of miR-146a expression and silencing of TRAF-6 resulted in a significant reduction in stress-induced cytokine secretion, suggesting that miR-146a targets the potential role of TRAF-6 in airway inflammation^15^.

Consistent with previous studies, our data showed that overexpression of miR-146a inhibited the inflammation process by targeting TRAF-6. To further investigate the potential mechanism of miR-146a related to the anti-inflammatory effect of RSV, the protein expression levels of possible TRAF-6 targets were detected, and it was found that the anti-inflammatory effect of miR-146a was accompanied by the inhibition of activation of JNK/ERK/MAPK/NF-κB pathways.

The study has shown that JNK1/2 is a key host factor for RSV production, and JNK activity is necessary for effective RSV production ^44^. Grape Seed Proanthocyanidin inhibits mucin synthesis and viral replication by suppression of AP-1 and NF-κB via p38 MAPKs/JNK signaling pathways in RSV-infected A549 cells^45^. Reduction of transepithelial electrical resistance induced by RSV infection was attenuated by p38 MAPK inhibitor, but was partially affected by JNK inhibitor, suggesting that RSV-induced human epithelial membrane permeability requires p38 MAPK^46^. ERK-1/2 activity is required for efficient RSV infection^47^, and activation of ERK2 by RSV in A549 cells is linked to the production of IL-8^48^. Functional investigations revealed that the late-stage activation of ERK is required for a specific step in RSV replication, namely, the secretory transport of the RSV fusion protein F^49^. Long-chain non-coding RNA n337374 relieves symptoms of respiratory syncytial virus-induced asthma by inhibiting dendritic cell maturation via the CD86 and the ERK pathway^50^. These data confirm the rich and complex role of the JNK/ERK/MAPK/NF-κB axis in RSV infection, and a large number of studies have revealed the regulatory role of miR-146a on these factors in different diseases. In colon cancer, miR-146a inhibits E-selectin expression by inhibiting the activity of NF-κB, a transcription factor of e-selectin, thereby reducing the adhesion and migration of colon cancer cells to and through endothelial cells^51^. By activating p38, ERK, and JNK and their downstream transcription factors GATA2, c-FOS, and c-Jun. Inhibition of p38 MAPK increased NF-κB activity, at least in part through miR-146a^51^. MiR-146a mimics ameliorate traumatic brain injury-related injuries via JNK and NF-κB signaling pathway^52^. TRAF-6 was identified as a target of miR-146a in human adipocytes, and decreased inflammatory JNK and p38 activation was detected after transfection with miR-146a^53^. In squamous cell carcinoma cells, miR-146A-5p and TRAF-6-specific siRNA down-regulated TRAF-6 inactivated JNK, but not in normal human keratinocytes^54^. Our results are the first to verify the regulation of miR-146a on TRAF-6 and the JNK/ERK/MAPK/NF-κB axis in RSV-infected cells and tissues.

In summary, our study found that the expression of miR-146a was significantly reduced in RSV-infected young rats and cells. Overexpression of miR-146a can target down-regulation of TRAF-6, reduce the release of inflammatory cytokines IL-1β, IL-6 and TNF-α, and reduce the inflammatory response and lung injury. The JNK/ERK/NF-κB/MAPK pathway may be a potential mechanism for miR-146a to exert an anti-RSV effect.

## Supplementary Information

Supplementary Figures.

## Data Availability

Data sharing not applicable to this article as no datasets were generated or analyzed during the current study.
