# Supplementary figures and images for: MiR-146a alleviates lung injury caused by RSV infection in young rats by targeting TRAF-6 and regulating JNK/ERKMAPK signaling pathways
Source: Sci Rep. 2022 Mar 3;12:3481. doi: 10.1038/s41598-022-07346-6 (PMC8894416; doi:10.1038/s41598-022-07346-6)

Figure 2D

Hep-2

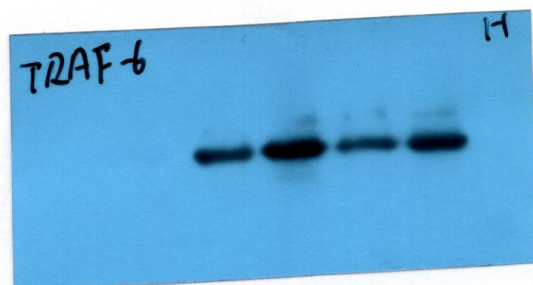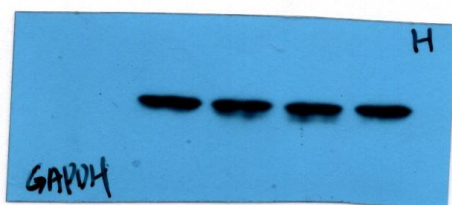

A549

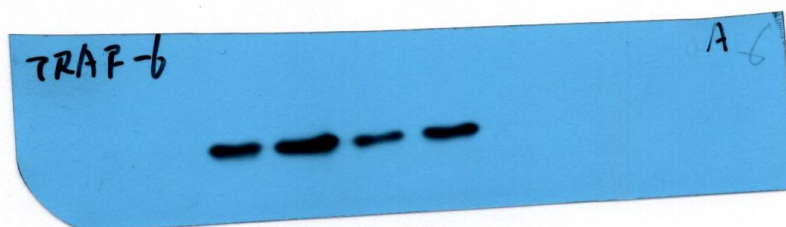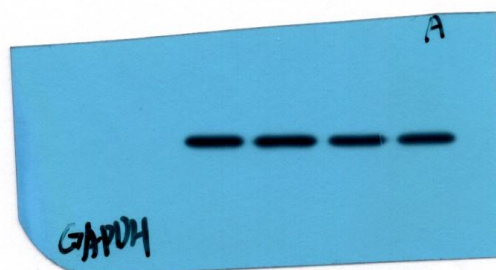

Figure 3B

A549

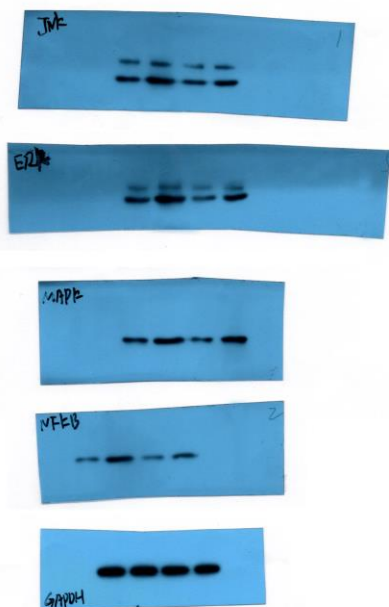

HEp-2

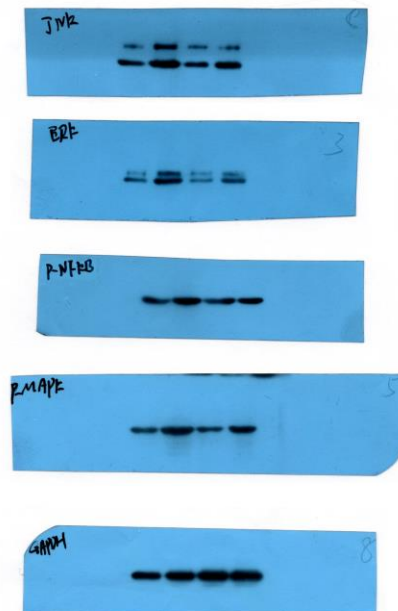

Figure 5C

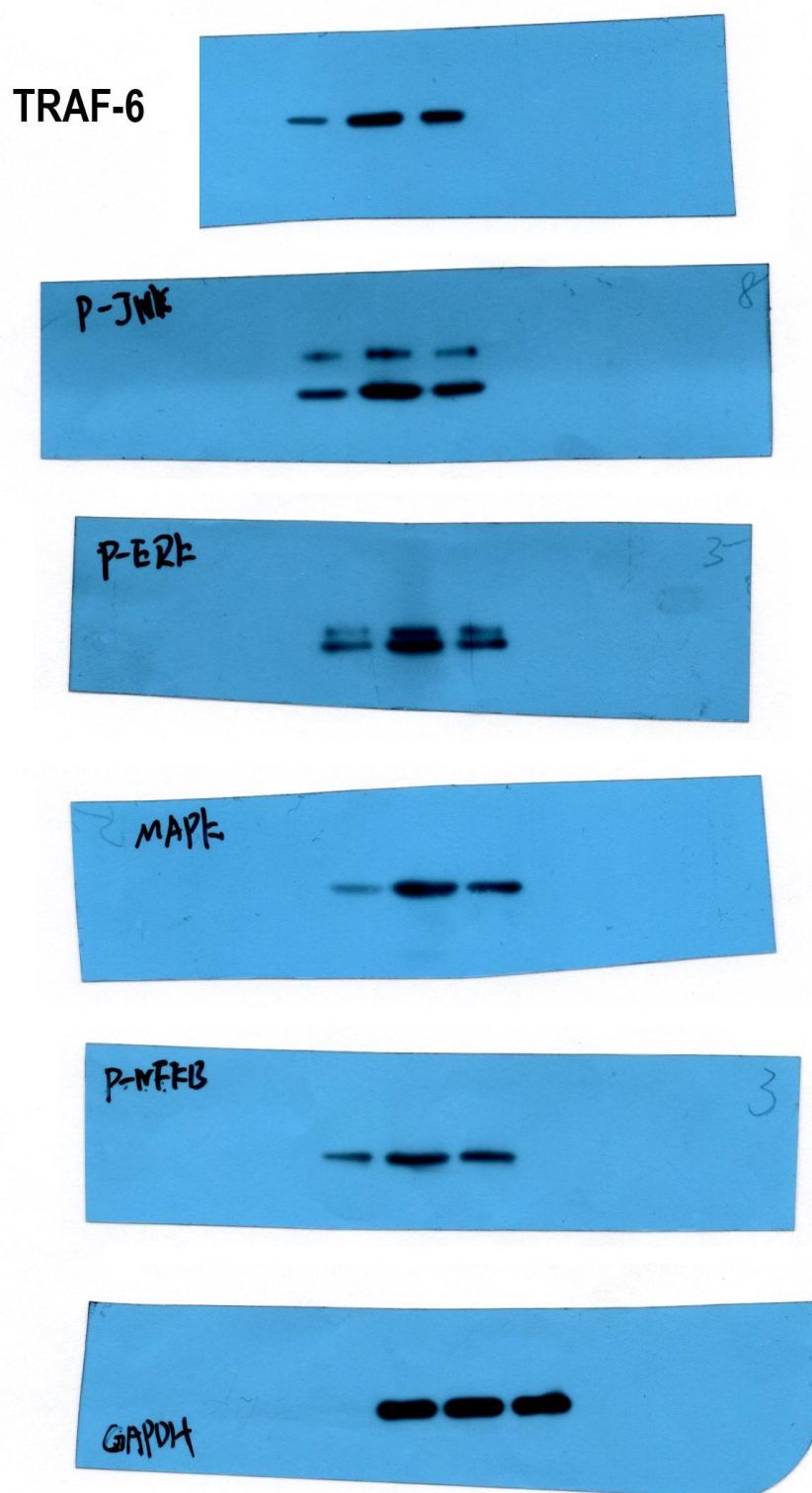

Supplement: Supplementary file 1 — Supplementary Figures. [file 41598_2022_7346_MOESM1_ESM.pdf]
